# Evaluation and Valorization of Ecological Risk Mitigation Through the Use of Sustainable Marine Resources in Ulva, a Marine Ecological Disturbance Species

**DOI:** 10.3390/biology14050551

**Published:** 2025-05-15

**Authors:** In-Yung Sunwoo, Yong-Kyung Ryu, Chul-Hong Oh, Woon-Yong Choi

**Affiliations:** Jeju Marine Research Center, Korea Institute of Ocean Science and Technology (KIOST), Jeju 63349, Republic of Korea; iysunwoo@kiost.ac.kr (I.-Y.S.); ykyou0111@kiost.ac.kr (Y.-K.R.); och0101@kiost.ac.kr (C.-H.O.)

**Keywords:** green algae bloom, seasonal dynamics, environmental responsiveness, costal ecosystem processes, sustainable ecosystem management

## Abstract

Massive blooms of *Ulva* species, commonly known as green tides, pose serious ecological threats by disrupting coastal ecosystems and requiring costly removal efforts. This study presents a nature-based solution by seasonally valorizing *Ulva ohnoi*, a bloom-forming macroalga dominant in Jeju Island, South Korea. Biomass was collected across all four seasons and subjected to phylogenetic identification, biochemical characterization, and bioresource processing. Despite environmental fluctuations, tufA-based analysis confirmed *U. ohnoi* as the sole species present year-round. Carbohydrate content peaked in spring (55.35%) and was lowest in summer (45.74%), corresponding to maximum reducing sugar of 36.49 g/L in winter and 36.24 g/L in spring following acid-enzymatic hydrolysis. The maximum ethanol fermentation using *Saccharomyces cerevisiae* produced up to 17.12 g/L ethanol in spring with a yield of 0.47 g/g. Post-fermentation residues were enzymatically hydrolyzed into Ulva Ethanol Residue Medium (UERM), which supported yeast growth and fermentation comparable to commercial YPD medium, achieving final optical densities of 8.3–8.5 and ethanol production of 16.5–16.8 g/L. Alanine, valine, and proline were the most abundant amino acids in UERM, supporting its suitability as a nitrogen source. These findings highlight the potential of integrating green tide mitigation with renewable energy and nutrient recycling through seasonal, localized biorefineries aligned with circular marine bioeconomy principles.

## Simple Summary

Green tides, caused by the excessive growth of certain macroalgae, are a serious threat to coastal ecosystems. In this study, we collected seaweed samples monthly for one year from the coastal waters of Jeju Island and identified all samples as *Ulva ohnoi* using genetic analysis. This species consistently dominated throughout the year, showing strong adaptability to seasonal changes in seawater temperature and salinity. We further evaluated the potential of *Ulva ohnoi* as a renewable bioresource by producing bioethanol and microbial culture media. Through acid-enzymatic pretreatment, high sugar yields were obtained, and stable ethanol production was achieved using yeast fermentation. Additionally, the residues left after fermentation were enzymatically hydrolyzed to produce Ulva Ethanol Residue Medium (UERM), which supported yeast growth and fermentation performance comparable to commercial media. This study demonstrates that *Ulva ohnoi* can not only contribute to ecological disturbance but also be transformed into a valuable resource, offering a sustainable approach to mitigating harmful algal blooms and promoting a circular bioeconomy.

## 1. Introduction

The frequent mass occurrence of *Ulva* species, commonly referred to as “green tides”, poses a growing ecological concern in coastal environments [1]. These blooms are often triggered by nutrient enrichment (eutrophication), elevated seawater temperatures, and hydrodynamic changes, which result in excessive macroalgal biomass accumulation along shorelines [2]. It is estimated that green tide removal efforts cost several million US dollars annually in severely affected coastal regions, highlighting the urgent need for effective and sustainable management strategies [3]. Such events disturb local marine biodiversity and generate environmental and economic burdens associated with biomass removal and disposal [4].

*Ulva ohnoi*, a bloom-forming green macroalga, has been frequently reported along the coastal areas of Jeju Island, South Korea, where it forms extensive mats that disrupt benthic habitats and contribute to coastal hypoxia [5]. Traditional approaches to manage macroalgal blooms, such as chemical treatments, mechanical removal, and dredging, often lead to secondary environmental damage, temporary effects, and high operational costs. These limitations necessitate the exploration of more ecologically compatible solutions [6].

Understanding the seasonal dynamics of the *Ulva* biomass and its biochemical traits is essential for evaluating its ecological functions and potential utilization [7,8]. Although numerous studies have addressed macroalgal blooms and their environmental impact, few have focused on how a stable taxonomic population of *Ulva* responds physiologically to seasonal environmental fluctuations such as changes in temperature, salinity, or nutrient availability. In particular, limited research has quantitatively assessed how seasonal environmental drivers influence the composition and valorization potential of *Ulva* biomass, which is critical for optimizing resource management strategies.

The sustainable use of macroalgal biomass has attracted increasing interest in recent years. The high carbohydrate content and rapid growth rate of *Ulva* make it a promising candidate for biofuel production and other biotechnological applications [9,10,11]. Commercial applications of *Ulva* include bioethanol and biogas production, high-protein animal feed, functional food ingredients, cosmetics, and fertilizers. *Ulva*-based biorefinery operations and food products have been successfully established in countries such as Japan, China, and parts of Europe, demonstrating its growing commercial relevance [12]. Harvesting *Ulva* during peak biomass periods may not only support circular bioeconomy initiatives but also serve as a nature-based strategy to mitigate the ecological risks associated with macroalgal overgrowth [13].

In this context, seasonal monitoring and strategic harvesting of *Ulva* sp. can offer the dual advantage of enabling timely biomass removal to prevent ecological degradation and providing a renewable, low-input feedstock for bio-based applications. Furthermore, repurposing post-fermentation residues for microbial media generation exemplifies a circular biorefinery strategy that ensures that each stage of biomass utilization contributes to waste minimization and nutrient recovery. This approach is particularly important because approximately 50–70% of seaweed biomass remains as solid residue after fermentation processes, offering significant opportunities for additional valorization [14]. This comprehensive framework aligns with the principles of nature-based solutions by addressing environmental challenges through ecologically compatible and socioeconomically beneficial technologies.

In this study, we aimed to evaluate the feasibility of valorizing *U. ohnoi* biomass collected across four seasons using a sequential biorefinery approach, including acid-enzymatic saccharification, ethanol fermentation, and protein hydrolysis of fermentation residues. By monitoring the seasonal variation in biochemical composition and linking it to biomass conversion efficiency, we sought to optimize the timing and application of biomass harvesting. By integrating this biomass utilization strategy with bloom management practices, we aimed to offer a comprehensive framework that simultaneously addresses coastal ecological concerns and advances in the development of renewable bioresources. This nature-based approach can contribute to the establishment of a low-carbon circular bioeconomy rooted in marine ecological engineering.

## 2. Materials and Methods

### 2.1. Seasonal Biomass Collection, Environmental Monitoring, and Species Identification via tufA Phylogeny

**Figure 1:**
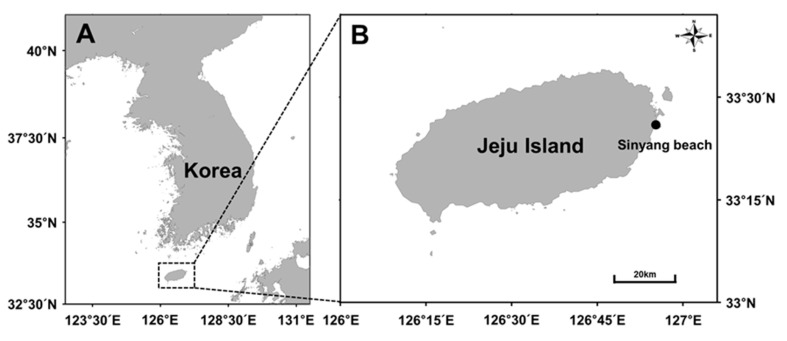
(**A**) Geographical location of Jeju island and (**B**) Detailed position of the sampling site (Sinyang Beach, Sungsaneup, Jeju, South Korea (33°25′52″ N, 126°55′31″ E)).

Biomass samples of *Ulva ohnoi* were collected monthly from the intertidal zone of Sinyang Beach, Sungsaneup, Jeju, South Korea (33°25′52″ N, 126°55′31″ E), over a 12-month period from March 2023 to February 2024 (Figure 1A,B). Sampling was conducted during low tide, once per month, with a total of 12 time points representing four seasons: spring (March–May), summer (June–August), autumn (September–November), and winter (December–February), as classified by the Korean Meteorological Administration (KMA). At each time point, approximately 300–500 g of fresh *Ulva* biomass was collected by hand using sterilized gloves and stainless-steel scissors. Care was taken to minimize disturbance to the surrounding habitat by collecting only loosely attached or floating thalli without deliberate classification by developmental stage. This limitation should be considered when interpreting biochemical variability, as age-related differences may influence cellular composition and hydrolysis efficiency [15]. Each sampling event was replicated three times at intervals of 5–10 m along a 30 m transect line parallel to the shoreline.

Environmental parameters, including sea surface temperature (°C) and salinity (psu), were measured in situ during each collection using a portable multi-parameter water quality meter (YSI 556 MPS, Yellow Springs Instruments, Yellow Springs, OH, USA). Air temperature data were obtained from the KMA database, referencing the nearest observation site in Sinyang beach, Seongsan-eup, Jeju.

The collected biomass was immediately rinsed with ambient seawater on-site to remove sediments, loosely adherent epiphytes, and macrofauna, followed by transportation to the laboratory in clean polyethylene bags on ice. In the laboratory, samples were gently washed again with distilled water, surface-dried with paper towels, and oven-dried using a far-infrared dryer (Garyeo, Siheungsi, Republic of Korea) at 50 °C for 48 h. Dried samples were ground using a household blender (Shinil, Cheonansi, Republic of Korea) and stored at 4 °C in airtight containers until further analysis.

For seasonal analyses, equal amounts of dried biomass from each month within a season were pooled to create representative composite samples. Each seasonal composite was prepared in triplicate to ensure analytical reproducibility. The triplicate preparations represent technical replicates of the same pooled biomass sample, ensuring reproducibility of the analytical procedures rather than assessing natural sample variation. To confirm the taxonomic identity of the collected *Ulva* biomass, genomic DNA was extracted from monthly samples collected between March 2023 and February 2024. The tufA gene was amplified by PCR and sequenced. A total of 12 sequences (one per month) were aligned with 12 reference tufA sequences of *Ulva* species obtained from GenBank. Multiple sequence alignment was performed, and phylogenetic relationships were inferred using the Maximum Likelihood (ML) method implemented in MEGA11 software [16].

The Tamura–Nei substitution model was applied to estimate evolutionary distances [17]. Initial trees for the heuristic search were generated automatically by applying Neighbor-Joining and BioNJ algorithms to a matrix of pairwise distances, and the final tree was selected based on the highest log likelihood value. The phylogenetic tree was drawn to scale, with branch lengths measured in the number of substitutions per site. A total of 24 nucleotide sequences and 1284 aligned positions were used in the final dataset.

Biochemical composition analysis of crude protein, lipids, ash, and fiber was conducted by the Institute of Agricultural Science, Chungnam National University (Republic of Korea) using official methods recommended by the Association of Official Analytical Chemists [18].

### 2.2. Pretreatment of Ulva Biomass

Ulva biomass collected from each season (spring, summer, autumn, and winter) was dried and subsequently ground into a fine powder before being subjected to a two-step saccharification process to obtain fermentable sugars. Briefly, biomass (10%, *w/v*) was treated with 1% (*v/v*) sulfuric acid (H_2_SO_4_) and autoclaved at 121 °C for 30 min. The hydrolysate was then neutralized to pH 6.0 using 10 N NaOH. For enzymatic saccharification, the hydrolysate was incubated with a cocktail of enzymes consisting of Celluclast 1.5 L (8 units/mL), Viscozyme L (1.2 FBG/mL), and Termamyl (120 KNU-T/g) (all from Novozymes, Bagsværd, Denmark) at 45 °C and 150 rpm for 12 h.

Thereafter, the samples were centrifuged at 8000 rpm for 10 min, and the supernatants were collected. The concentration of reducing sugars in each sample was quantified using the 3,5-dinitrosalicylic acid (DNS) method. The total amount of reducing sugars in each hydrolysate was estimated using a standard curve of glucose. The absorbance was measured at 540 nm using a spectrophotometer.

The efficiency of pretreatment (E_P_) was calculated to evaluate how effectively the carbohydrates in the biomass were converted into fermentable sugars after the acid and enzymatic treatments. The E_P_ value was calculated using the following equation:

(1) $$E_{P}=\frac{{\left[Reducing\;sugar\right]}_{After\;pretreatment}}{{\left[Carbohydrate\;content\;in\;biomass\right]}_{initial}}\times 100$$

Here, “reducing sugar concentration after pretreatment (g/L)” refers to the total amount of reducing sugars released from the biomass following acid and enzymatic hydrolysis, as measured by the DNS method, and “initial carbohydrate content in the biomass (g/L)” refers to the total carbohydrate proportion originally measured in the dry biomass prior to pretreatment. The E_P_ value reflects the conversion efficiency of structural carbohydrates into fermentable sugars, with higher values indicating a more effective pretreatment process [19].

### 2.3. Ethanol Fermentation Usign Sacchromyces cerevisiae

Fermentation was carried out in a 250 mL Erlenmeyer flask with a working volume of 100 mL. The yeast *Saccharomyces cerevisiae* KCTC 1126 was used for fermentation. The inoculum was added to the hydrolysate at a concentration of 0.2 g dcw/L. The fermentation was carried out at 30 °C and 150 rpm for 144 h. Samples were taken periodically for analysis. The ethanol yield (Y_EtOH_) of the samples was calculated using the following equation:

(2) $$Y_{EtOH}=\frac{{\left[EtOH\right]}_{Max}}{{\left[Reducing\;sugar\right]}_{initial}}$$

where Y_EtOH_ is the ethanol yield (g/g), [EtOH]max is the highest ethanol concentration achieved during fermentation (g/L), and [monosaccharide]ini is the total initial sugar concentration at the onset of fermentation (g/L) [8]. The highest theoretical Y_EtOH_ is 0.51.

### 2.4. Enzyme Hydrolysis and Amino Acid Analysis in Ulva Ethanol Residue (UER)

After ethanol fermentation, the remaining biomass residue (UER) was collected, dried, and ground to powder. The UER was subjected to enzymatic hydrolysis to release protein-derived nitrogen sources for the development of microbial media. The UER powder was dissolved in distilled water at 1% (*w/v*), and the pH was adjusted to 7.0. Thereafter, protease (Sigma-Aldrich, St. Louis, MO, USA) was added a 1% (*w/v*) concentration, and the reaction mixture was incubated at 60 °C for 24 h with shaking at 150 rpm.

To inactivate the enzyme, the hydrolysate was heated at 95 °C for 10 min. The mixture was then centrifuged at 7000 rpm and 4 °C for 30 min. The supernatant (aqueous soluble fraction) was collected and freeze-dried to obtain *Ulva* ethanol residue medium (UERM) powder, which was further characterized.

Total amino acid content was determined by hydrolyzing the sample with 6 N HCl at 130 °C for 24 h, followed by HPLC analysis (Agilent 1200 LC, Santa Clara, CA, USA). Free amino acids were extracted using perchloric and metaphosphoric acids and analyzed using a Dionex UHPLC3000 amino acid analyzer (Sunnyvale, CA, USA). Table 1 summarizes the chromatographic conditions. For free amino acid analysis, 1 g of the sample was mixed with 0.1% perchloric acid and 0.1 M metaphosphoric acid, followed by the addition of triple-distilled water to obtain a final volume of 50 mL. The mixture was subjected to ultrasonic extraction (WUC-D22 H; Daehan Scientific Co., Ltd., Wonjusi, Republic of Korea) for 60 min, followed by additional extraction at room temperature for 60 min. The supernatant was filtered through a 0.2 µm syringe filter and analyzed using an amino acid analyzer (Dionex UHPLC3000 systems, Sunnyvale, CA, USA) under the conditions specified in Table 1.

### 2.5. Yeast Growth Evaluation in Ulva Ethanol Residue Medium (UERM)

To evaluate the efficacy of UERM as a nitrogen source for microbial cultivation, the growth performance of *S. cerevisiae* was assessed in UERM-containing media and compared to standard peptone-based media.

Culture media were prepared by adding 10 g/L peptone (either UERM, animal-derived, or plant-derived), 5 g/L yeast extract, and 20 g/L glucose to distilled water. Commercial animal and plant peptones were purchased from BD DifcoTM, whereas UERM was prepared from an enzymatically hydrolyzed *Ulva* ethanol residue, as described in Section 2.2.

Yeast cells were pre-cultured in YPD (10 g/L yeast extract, 20 g/L peptone, and 20 g/L glucose) overnight and then inoculated into the experimental media at an initial OD600 of 0.1. Cultures were incubated at 30 °C with shaking at 150 rpm for 48 h. Real-time growth monitoring was performed using a Real-Time Cell Growth Logger (Biosan, Riga, Latvia), which measured OD600 every 20 min during the cultivation period.

### 2.6. Assessment of Fermentation Efficiency Using UERM Cultured Yeast 

To assess the fermentation competency of yeast cultivated in the UERM, ethanol production was tested using *Ulva*-derived sugar hydrolysate as the fermentation substrate.

After 24 h of growth in each medium (UERM or YPD), *S. cerevisiae* cells were harvested, washed, and inoculated into 100 mL of *Ulva* hydrolysate prepared via acid pretreatment and enzymatic saccharification, as described in Section 2.2. Fermentation was carried out in 250 mL Erlenmeyer flasks at 30 °C and 150 rpm for 24 h.

Ethanol concentrations were measured at the end of fermentation using HPLC, and the fermentation efficiency was compared based on the final ethanol yield (g/L) and relative fermentation rates.

### 2.7. Statistical Analysis

All experimental data were analyzed in IBM SPSS Statistics (ver. 23.0; SPSS Inc., Chicago, IL, USA). Statistical significance among the experimental groups was evaluated using one-way analysis of variance (ANOVA), followed by Duncan’s multiple range test (DMRT) as a post hoc analysis. Duncan’s test was used to determine significant differences between mean values at a confidence level of *p* < 0.05. All results are presented as mean ± standard deviation (SD) of at least three independent replicates.

## 3. Results

### 3.1. Seasonal Variations in Environmental Conditions, Phylogenetic Confirmation, and Biomass Composition of Ulva Biomass

**Figure 2:**
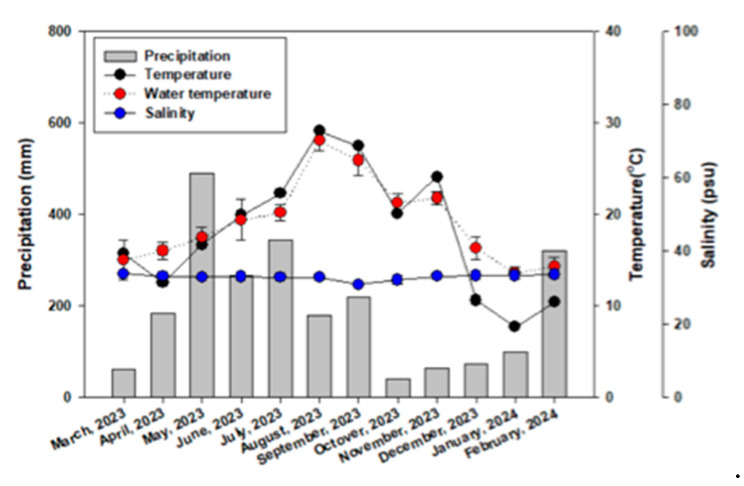
Seasonal variations in environment parameters.

Environmental conditions exhibited clear seasonal patterns (Figure 2). Water temperature ranged from 13.6 °C in winter to 28.1 °C in summer, whereas air temperature fluctuated between 7.7 °C and 29.1 °C. Precipitation was the highest during the summer season, peaking in May (489.8 mm). Salinity remained relatively stable, varying slightly from 30.8 to 33.8 ppt, with minor decreases observed during the summer season when rainfall was high.

**Figure 3:**
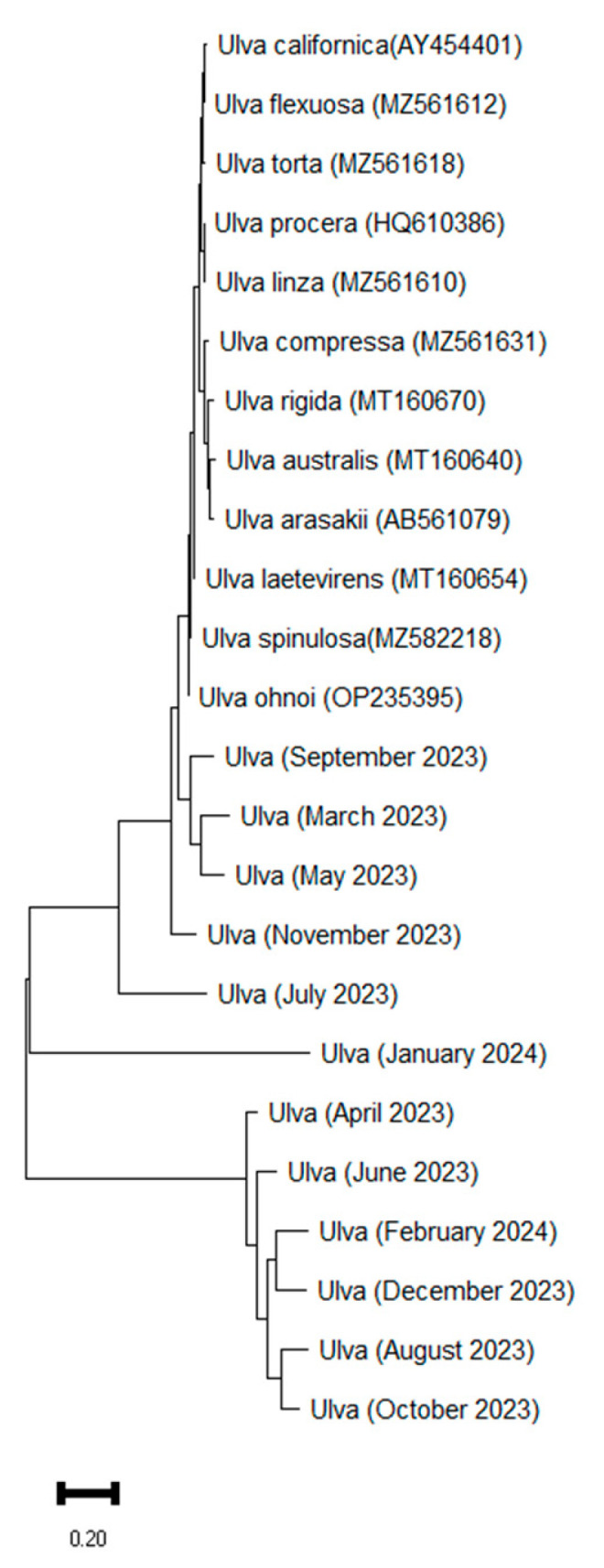
Maximum likelihood (ML) phylogram tree based on tufa sequences of 12 *Ulva* samples collected from Sinyang, Jeju.

The phylogenetic analysis confirmed that all 12 monthly *Ulva* samples clustered tightly within the *Ulva ohnoi* clade (GenBank accession: OP235395), along with high sequence similarity across the year (Figure 3). No other *Ulva* species were detected, indicating taxonomic consistency and species-level dominance of *U. ohnoi* throughout the sampling period.

The Maximum Likelihood tree, constructed using the Tamura–Nei model, yielded the highest log likelihood score of −25,513.61. The resulting tree showed that the seasonal samples formed a robust monophyletic group with *U. ohnoi*, clearly separated from closely related taxa such as *U. compressa*, *U. flexuosa*, and *U. spinulosa*. These findings confirm the year-round ecological dominance of *U. ohnoi* in the coastal environment of Jeju and validate the suitability of the tufA marker for species-level resolution in macroalgal biodiversity studies.

In contrast, significant seasonal differences were observed in the biochemical composition of biomass (Table 2). Carbohydrate content was highest in spring (55.35 ± 3.04%), followed by autumn (53.31 ± 2.40%), winter (50.39 ± 1.58%), and lowest in summer (45.74 ± 3.90%). Protein content peaked in winter (21.64 ± 1.87%) and was the lowest in autumn (11.62 ± 0.56%). Ash content also followed a similar pattern, with the highest value in winter (28.6 ± 2.7%). Crude lipid levels remained consistently low (<1.5%) in all seasons, whereas fiber content was slightly higher in autumn and winter.

These results suggest that although environmental conditions did not influence species composition, they strongly influenced biomass composition. The consistency of *U. ohnoi* year-round, despite seasonal fluctuations in its biochemical composition indicated a high degree of physiological plasticity in this species.

### 3.2. Sugar Yield and Hydrolysis Efficiency of Seasonal Ulva Biomass

**Figure 4:**
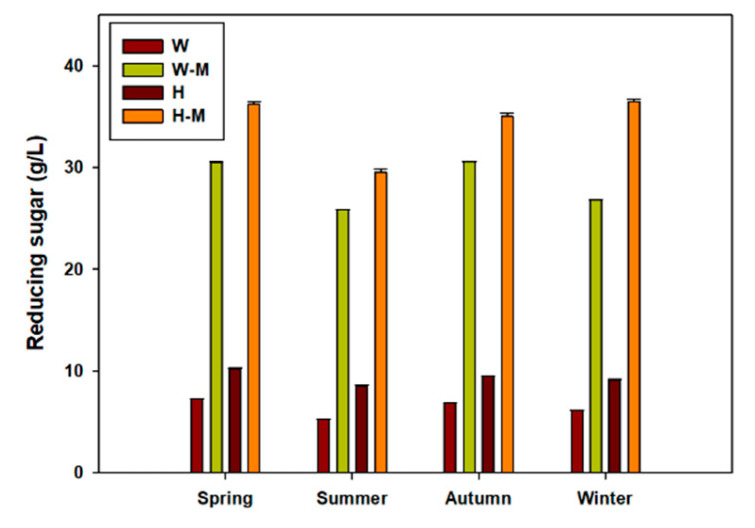
Seasonal variations in reducing sugar yield and pretreatment efficiency of *Ulva* biomass (W, water extraction only; W-M, water extraction followed by enzymatic saccharification; H, acid hydrolysis (1% H_2_SO_4_); H-M, acid hydrolysis followed by enzymatic saccharification), Bar represent means ± SD (*n* = 3).

The yield of reducing sugars, obtained from the acid and enzymatic hydrolysis of *U. ohnoi* biomass, exhibited clear seasonal variations, reflecting changes in biomass composition and the response of each seasonal sample to pretreatment. Using the DNS method, the total reducing sugar content was quantified after combining 1% H_2_SO_4_ pretreatment and enzymatic saccharification (Figure 4).

The highest sugar yields were obtained from winter and spring samples, with average values of 36.49± 0.24 g/L and 36.24 ± 0.25 g/L, respectively. These samples had relatively high carbohydrate contents (winter: 50.39%, spring: 55.35%), responded well to the hydrolysis process, and showed favorable responsiveness to acid-enzymatic pretreatment. The corresponding E_P_ values for winter and spring samples were 72.42% and 65.48%, respectively.

The autumn sample yielded 35.20 ± 0.35 g/L of monosaccharides, with a carbohydrate content of 53.31% and an E_P_ value of 65.67%, suggesting moderate conversion efficiency. In contrast, the summer biomass, which had the lowest carbohydrate content (45.74%), also exhibited the lowest sugar yield (29.52 ± 0.32 g/L) and hydrolysis efficiency (E_P_ values: 64.55%). This result indicates a compound effect of reduced fermentable substrate availability and lower pretreatment and saccharification efficiency during summer.

**Figure 5:**
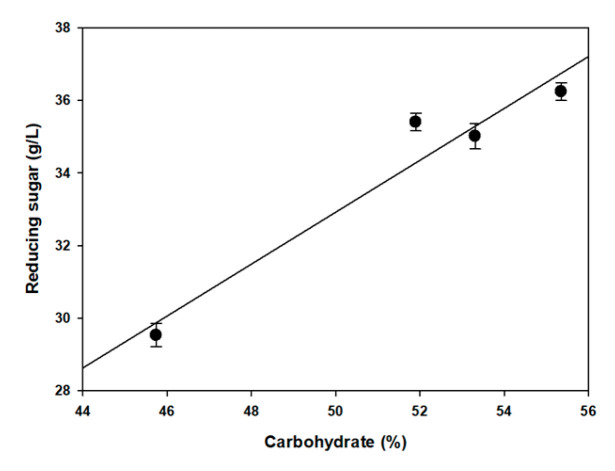
Relation of reducing sugar and carbohydrate.

The overall sugar yield followed the same trend as the measured seasonal carbohydrate content: winter ≈ spring > autumn > summer. Pearson’s correlation analysis confirmed a strong relationship between the carbohydrate content and sugar yield (r^2^ = 0.94, *p* < 0.05), suggesting that the initial biomass composition remains the primary driver of sugar productivity under consistent processing conditions (Figure 5).

DNS identified glucose as the dominant reducing sugar in all samples, which was consistent with the known saccharide composition of *Ulva* species. However, differences in the structural composition of polysaccharides, such as ulvan or cellulose content, may have contributed to the variability in the response to acid and enzymatic treatments across seasons.

### 3.3. Ethanol Production of Ulva Biomass Across Seasons

**Figure 6:**
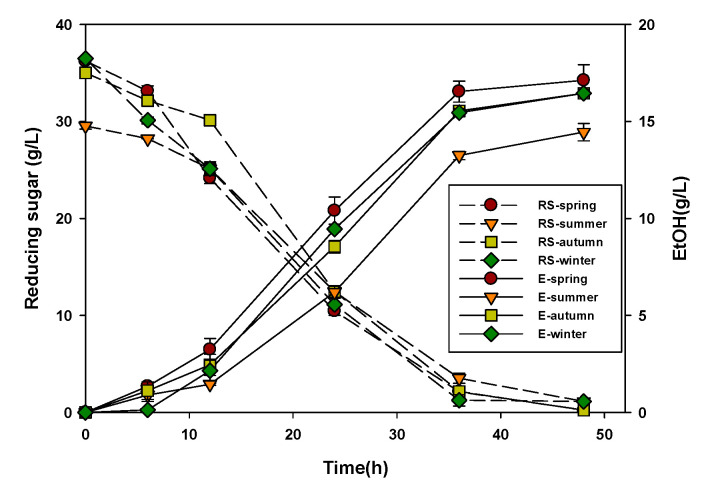
Seasonal variations in reducing sugar (RS) and ethanol concentration (E) in *Ulva* biomass hydrolysates.

Figure 6 shows the seasonal differences in reducing sugar yield, final ethanol production, and ethanol yield (Y_EtOH_).

The spring hydrolysate sample yielded the highest ethanol concentration of 17.12 g/L after 48 h of fermentation. This corresponded to an ethanol yield of 0.47 g/g, which was the highest among all samples across seasons. The winter sample, although containing a slightly lower initial reducing sugar (32.50 g/L), produced a comparable ethanol concentration of 15.45 g/L with an ethanol yield of 0.48 g/g. Spring and winter samples also demonstrated the highest pretreatment efficiency, with E_P_ values of 72.42% and 65.48%, respectively, indicating the effective conversion of biomass carbohydrates into fermentable sugars.

In contrast, the summer hydrolysate resulted in the lowest ethanol production (13.45 g/L) and yield (0.46 g/g) despite being processed under identical conditions. The E_P_ value for summer (64.55%) was also the lowest among all the groups, suggesting that pretreatment and saccharification process are less efficient in summer. The autumn biomass displayed intermediate performance, yielding 16.45 g/L ethanol and 0.47 g/g with an E_P_ value of 65.67%.

Overall, ethanol yields from all seasonal samples were relatively high, ranging from 0.46 to 0.48 g/g, equivalent to 90–94% of the theoretical maximum yield (0.51 g/g). These results indicated that *U. ohnoi* is a suitable bioresource for ethanol production throughout the year, with clear seasonal differences in conversion efficiency.

### 3.4. Amino Acid Composition of UERM Derived from Seasonal Biomass

Seventeen amino acids were identified and quantified from each seasonal UERM sample (Table 3). Alanine, valine, and proline were consistently ranked as the top three amino acids in all seasonal samples, accounting for approximately 43–45% of the total amino acids. In the spring UERM, alanine was the most abundant (1.57%), followed by valine (1.31%) and proline (1.20%). The autumn and winter samples showed similar trends, with alanine levels ranging from 1.46% to 1.52%, and valine levels ranging from 1.21% to 1.23%. Summer UER had a slightly lower total amino acid content.

Other amino acids, such as phenylalanine, lysine, and methionine, were detected at moderate levels, each contributing between 6.5 and 7.5% of the total amino acids, with seasonal fluctuations. Hydrophilic amino acids, including aspartic acid, glutamic acid, and glycine, which are typically abundant in native *Ulva* biomass, were detected at relatively low levels (<5%) regardless of the season.

Notably, the total amino acid content was highest in the spring UERM sample, followed by winter and autumn samples, with the lowest content observed in the summer samples.

### 3.5. Evaluation of Yeast Growth and Fermentation in UERM

Growth kinetics of *S. cerevisiae* cultured in UERM were compared with those cultured in the YPD medium, and fermentation performance was evaluated using *Ulva*-derived sugar hydrolysate.

#### 3.5.1. Yeast Growth Dynamics in Seasonally Derived UERM

**Figure 7:**
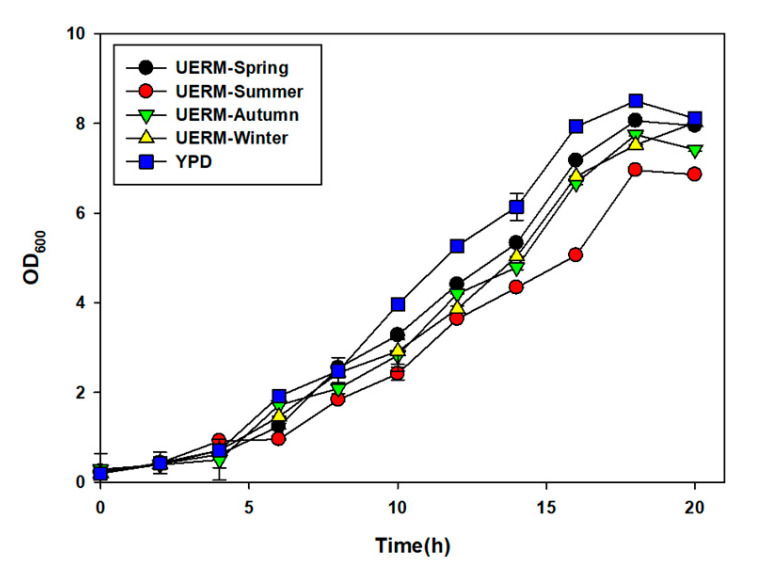
Comparison of yeast growth profiles in UREM and commercial YPD medium.

Yeast growth was successfully supported in UERM media in all samples across seasons, with final biomass concentrations being comparable to those in YPD (Figure 7). The spring UERM facilitated the fastest initial growth rate of yeast, with OD600 exceeding 8.0 after 12 h and reaching the stationary phase by 18 h.

In the autumn and winter UERM, the growth curve of yeast displayed a longer lag phase but ultimately attained a final OD comparable to that of YPD. Summer UERM faciliated the slowest growth rate, with a delayed exponential phase entry and marginally reduced final biomass. However, all UERM media, regardless of the season, supported overall yeast proliferation to a level statistically equivalent to that of YPD-grown cells (*p* > 0.05).

#### 3.5.2. Ethanol Yield from UERM Cultured Yeast 

To evaluate the impact of UERM on fermentation performance, *S. cerevisiae* cells grown in each seasonal UERM were used to ferment the *Ulva*-derived monosaccharide hydrolysate (Table 4). After 24 h of fermentation, the spring-grown yeast produced the highest ethanol concentration (16.83 g/L), followed by the winter-grown yeast (16.52 g/L). The autumn and summer groups yielded 15.73 g/L and 12.94 g/L, respectively, showing no statistically significant difference compared to YPD-grown yeast (13.45–17.12 g/L).

In terms of the fermentation rate, the initial ethanol production was more rapid in yeast cells cultivated in spring and winter UERM, which was consistent with the higher early-stage growth rates observed during cultivation. This likely reflects greater accumulation of active biomass prior to fermentation, contributing to enhanced sugar conversion during the initial phase.

Despite minor differences in the early kinetics, all groups achieved similar final ethanol concentrations, indicating that UERM supports a fermentative capacity comparable to that of conventional media. These results demonstrate that across all seasons, UERM provides adequate nutritional support for both yeast growth and ethanol production.

## 4. Discussion

### 4.1. Ecophysiological Insight and Taxonomic Consistency of Ulva ohnoi Across Seasons

*U. ohnoi* was consistently identified as the sole dominant species throughout all four seasons, confirming its ecological dominance, taxonomic stability, and adaptability to eutrophic coastal environments, including those in Jeju Island. Despite substantial seasonal fluctuations in sea surface temperature and other environmental parameters, this species maintained its presence, underscoring its physiological plasticity and resilience. Our results also indicated that the use of tufA as a molecular marker was effective in distinguishing *U. ohnoi* from closely related taxa, highlighting its value for seasonal and regional biodiversity monitoring. Compared with other commonly used markers such as rbcL, the tufA gene offers higher phylogenetic resolution and greater discriminatory power within the genus *Ulva*, making it especially useful for resolving taxonomic ambiguities among morphologically similar species [5].

The average carbohydrate content of *Ulva ohnoi* biomass analyzed in this study across four seasons was 51.20%, which aligns well with previously reported values for various *Ulva* species. Although slightly higher than the average carbohydrate content (50.65%) reported for various *Ulva* species in previous studies, the seasonal mean of *U. ohnoi* biomass obtained in this study (51.20%) remains within the established range (43.0–54.3%) [20]. This suggests that *U. ohnoi* possesses a comparable, if not superior, carbohydrate profile for bioconversion applications. Its consistent performance across seasons underscores its potential as a reliable macroalgal feedstock for sustainable biofuel production.

The biochemical plasticity observed in *U. ohnoi* across the seasons highlights its ecophysiological adaptability. Contrary to the general assumption that macroalgae accumulate more carbohydrates during summer because of increased photosynthetic activity, our results showed that the carbohydrate content was highest in spring and lowest in summer. The increase in protein content and the corresponding decrease in carbohydrate proportion summer may be attributed to the increase in nitrogen availability from nutrient runoff in early summer, which is driven by rainy season; this increased nitrogen availability could shift metabolic allocation toward protein synthesis. Additionally, during summer, when *U. ohnoi* is likely undergoing its most rapid vegetative growth, the products of photosynthesis are immediately utilized for biomass expansion and cellular construction rather than being stored as polysaccharides [21]. This pattern is consistent with the growth-over-storage strategy, in which algae prioritize active proliferation over carbohydrate accumulation during peak growth periods [22].

In contrast, spring may represent a pre-growth storage phase, wherein photosynthates are stored in preparation for the high metabolic demands of summer. Our finding of low protein content in spring further supports this assumption as nitrogen assimilation during this season may be relatively limited, allowing a greater proportion of carbon to be retained as carbohydrates [23].

The winter biomass profile, characterized by elevated protein and ash contents, suggests a shift toward nutrient retention and stress tolerance, potentially reflecting slowed growth under colder, low-light conditions [8]. These compositional shifts reveal seasonally adaptive metabolic strategies in *U. ohnoi*, which are optimized for environmental conditions.

From an application standpoint, spring-harvested biomass, with its high carbohydrate content, is well suited for bioethanol production, whereas winter and summer biomasses, which are richer in protein and ash, are more appropriate for microbial media development or nutrient recycling [24]. This information supports the development of seasonally targeted harvesting strategies within a circular bioeconomy framework.

In conclusion, *U. ohnoi* demonstrates clear functional flexibility in response to environmental changes, despite its taxonomic uniformity throughout the year. Recognizing and utilizing this decoupling between species identity and biochemical function is the key to both ecological monitoring and industrial resource optimization in macroalgal biorefineries.

### 4.2. Impact of Seasonal Traits on Hydrolysis and Sugar Recovery

The observed seasonal differences in sugar yield and hydrolysis efficiency reflected the complex interplay between biomass composition and structural accessibility. Winter and spring samples exhibited high carbohydrate content and effective hydrolysis, yielding the highest sugar levels. However, summer biomass was the least efficient in terms of absolute sugar conversion and carbohydrate-to-sugar conversion rate (E_P_). The low E_P_ value in summer (64.55%), combined with its low carbohydrate content, suggests that the biomass harvested during this period may possess denser or more recalcitrant cell wall structures, potentially due to rapid growth and seasonal physiological changes [25]. These changes may lead to an increased accumulation of structural polysaccharides (such as ulvan) or enhanced mineral binding, both of which can inhibit acid penetration and enzymatic action [26].

In contrast, winter biomass not only contained substantial carbohydrate reserves but also responded well to hydrolysis (E_P_ value: 72.42%), likely due to reduced metabolic activity and softer tissue structure associated with slower growth under colder conditions [27].

The autumn and spring samples exhibited intermediate characteristics, with comparable carbohydrate levels and moderate hydrolysis performance. These results emphasize that higher sugar yield is not solely determined by carbohydrate content but also by the digestibility and structural openness of the biomass, which are seasonally regulated.

From industrial applications, spring and winter harvests appear to be most suitable for maximizing fermentable sugar output, whereas summer-harvested biomass may require process optimization, such as increased acid concentration or longer enzymatic hydrolysis, to overcome its reduced reactivity [28]. This suggests that seasonally adjusted biorefinery strategies can enable a more efficient and sustainable utilization of marine macroalgal resources.

### 4.3. Influence of Biomass Seasonality on Ethanol Production 

Ethanol production from *U. ohnoi* hydrolysates exhibited seasonal variations, providing insights into how biomass characteristics, pretreatment responsiveness, and sugar composition dynamically interact to influence biofuel yield.

The superior ethanol output from the spring and winter samples reflects both higher initial sugar concentrations and more efficient hydrolysis, as evidenced by the elevated E_P_ values. Biomass harvested during these periods likely exhibited enhanced accumulation of storage polysaccharides (such as starch-like glucans) and reduced structural rigidity owing to slower growth rates and colder environmental conditions [29]. These features may contribute to greater enzyme accessibility and faster sugar release, facilitating complete and rapid fermentation by *S. cerevisiae*.

Interestingly, spring biomass had the highest carbohydrate content, its ethanol yield (0.47 g/g) was slightly lower than that of winter biomass (0.48 g/g). This suggests that protein and mineral content, often elevated in winter samples, could support yeast metabolism during fermentation, partially explaining the higher yield obtained from winter hydrolysates despite lower initial sugar levels [30]. In contrast, the summer biomass resulted in the lowest ethanol yield and concentration, indicating possible structural or biochemical constraints. The reduced E_P_ value (64.55%) of summer biomass indicates either a lower saccharification efficiency or greater retention of sugars in recalcitrant forms, such as ulvan-rich matrices or sulfated polysaccharides that are less digestible [19]. Additionally, the possibility of accumulation of inhibitory compounds, such as phenolics, salts, or organic acids, during active summer cannot be ruled out; this accumulation may suppress yeast viability or metabolic rate during fermentation [31].

The autumn sample, which showed intermediate behavior in both hydrolysis and fermentation, further supports the idea of seasonal metabolic transition, possibly reflecting a shift from growth-focused to storage-focused biomass as temperatures reduce [32].

Despite these differences, all samples achieved 90–94% of the theoretical ethanol yield, confirming the year-round potential of *U. ohnoi* as a bioresource. In comparison with previous studies on macroalgal ethanol production, the ethanol yield obtained from *Ulva* ohnoi hydrolysates in this study (0.47–0.48 g/g, or 90–94% of the theoretical maximum) demonstrates a competitive performance. For example, *Ulva fasciata* yielded 0.42 g/g (88% efficiency), while red algae like *Gracilaria verrucosa* and *Gelidium amansii* achieved comparable yields only after thermal pretreatment or prolonged fermentation. Moreover, several red and brown algae (*Kappaphycus alvarezii, Sargassum* spp.) demonstrated lower ethanol yields (0.29–0.35 g/g) or required thermophilic or co-culture fermentation strategies, which may be less feasible for large-scale implementation [20].

In contrast, the efficient hydrolysis and fermentation of *U. ohnoi* within 48 h under standard SHF conditions, without the need for genetic modification or thermal pretreatment, highlights its operational simplicity and economic viability. This underscores the potential of *U. ohnoi* as a robust, green macroalgal resource for sustainable bioethanol production. Integrating seasonal biomass dynamics into biorefinery planning will further improve feedstock reliability and process efficiency, supporting circular marine bioeconomy goals.

### 4.4. Amino Acid Profile of UERM and It’s Seasonal Variability

Notable differences were observed in the composition and abundance of individual amino acids among the samples from the four seasons, reflecting the physiological characteristics of the biomass and enzymatic digestibility of its protein components.

Across all seasons, alanine, valine, and proline were the most abundant amino acids, accounting for more than 40% of the total amino acid content. These amino acids are readily metabolized by *S. cerevisiae* and play important roles in microbial growth and stress tolerance [33]. In a previous study on *Ulva* sp., the protease Pronase exhibited the highest hydrolysis efficiency (95%) compared to pancreatin (67%) and pepsin (17%),, indicating that the enzymatic protocol employed was highly effective in liberating amino acids relevant to microbial metabolism [34]. These results highlight the suitability of Pronase for breaking down complex seaweed proteins into bioavailable forms, particularly for use in microbial cultivation media.

Notably, spring-derived UERM exhibited the highest total amino acid concentration, followed by winter-, autumn-, and summer-derived UERM. The superior amino acid yield observed in spring may be attributed to increased protein accumulation during the early growth phase of *U. ohnoi*, when metabolic activity and nutrient uptake peak [35]. In contrast, the lower amino acid content in summer samples is likely due to the physiological stress associated with higher temperatures and light intensity, which may lead to increased synthesis of recalcitrant structural proteins or protective compounds that are less amenable to enzymatic degradation [36].

Several hydrophilic amino acids commonly reported as major constituents of *Ulva* species, such as glutamic acid, glycine, and aspartic acid, were present in relatively low concentrations in all UERM samples [37]. This discrepancy is likely attributable to the high solubility of these amino acids, making them prone to loss during ethanol extraction and washing or their potential degradation during hydrothermal processing [35]. These observations underscore the need for process optimization to minimize the loss of labile amino acids during processing.

From a biotechnological perspective, the amino acid composition of the UERM indicates its strong potential for use as a low-cost and sustainable nitrogen source in microbial culture media. The presence of high levels of assimilable amino acids, particularly in the spring and winter samples, suggests that UERM could partially or fully substitute commercial peptone or yeast extract in yeast fermentation systems.

In summary, the UERM derived from *U. ohnoi* residues presents promising characteristics as a protein hydrolysate-based medium for microbial fermentation. However, seasonal variations in the amino acid profile and overall yield should be considered when designing consistent and efficient fermentation processes. Further investigations into enzyme combinations, hydrolysis conditions, and amino acid recovery strategies are warranted to maximize the nutritional and functional values of Ulva-derived fermentation residues for circular bioeconomy applications.

### 4.5. UERM as Sustainable Nitrogen Source: Performance and Application Potencial

The growth rate and ethanol production performance of *S. cerevisiae* cultured in UERM was comparable to those observed for *S. cerevisiae* cultured in standard YPD medium. This indicates the viability of UERM as an effective alternative nitrogen source for yeast-based bioprocesses. Despite the absence or low abundance of certain amino acids (histidine, cysteine, and tryptophan) in the UERM amino acid profile, the yeast cells showed no observable growth or fermentation limitations. This is likely because *S. cerevisiae* is capable of de novo synthesis of these amino acids under normal culture conditions [38].

Though glutamic acid contributes significantly to nitrogen metabolism and stress resilience in yeast, its relatively low abundance in UERM (approximately 4%) did not negatively affect yeast growth [39]. This observation is supported by the high levels of alanine, valine, and proline in UERM, which are not only readily assimilated, but also play functional roles in stress tolerance, osmoregulation, and energy metabolism [40]. Proline and valine are associated with oxidative stress mitigation and membrane stabilization, which may contribute to the enhanced early-phase growth and robust fermentation performance observed in the present study.

The fermentation performance further validated the functional robustness of the UERM. The similar final ethanol concentrations across all media, along with the higher initial production rate in UERM-grown cultures, indicated that UERM does not impair yeast fermentative capacity. In contrast, it may enhance early metabolic activity by supporting faster yeast biomass build-up and improving stress resistance [39].

This study is subject to several limitations. The sampling was geographically restricted to a single coastal site, and only *Ulva ohnoi* was identified throughout the year, limiting insights into broader species dynamics. The biorefinery process was evaluated under laboratory-scale conditions using a single acid-enzymatic pretreatment strategy, without comparison to alternative methods or economic feasibility assessment. Future research should include multi-site sampling, explore diverse pretreatment and fermentation approaches, and integrate techno-economic and life cycle assessments. Pilot-scale validation will be essential to support the practical implementation of seasonal *Ulva* biomass valorization in coastal ecosystem management.

**Figure 8:**
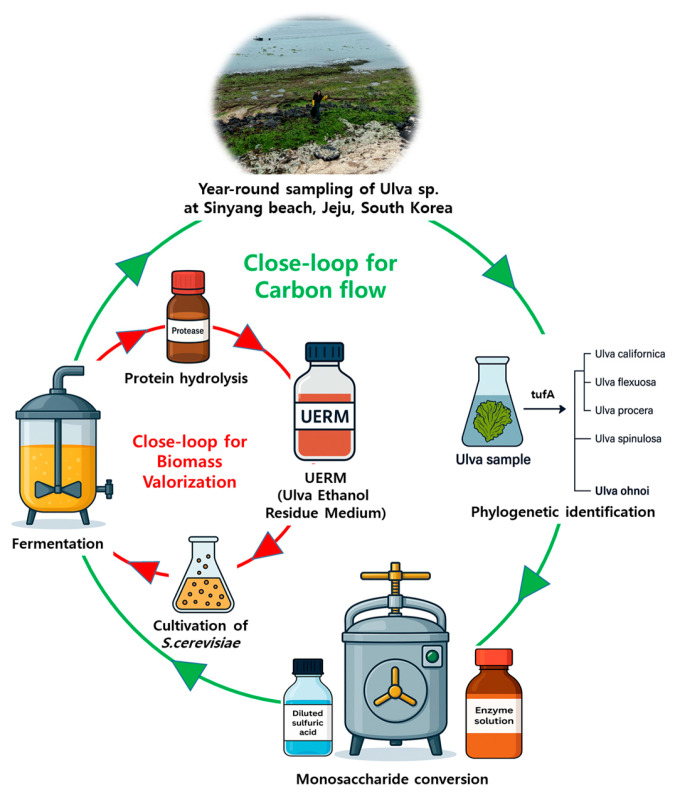
Integrated Valorization of *Ulva ohnoi*: Biomass and Carbon Cycling in a Marine Biorefinery Framework.

In addition to demonstrating the seasonal viability of *Ulva ohnoi* for bioethanol production and microbial media development, this study illustrates a dual closed-loop framework encompassing both biomass and carbon cycling (Figure 8). The first loop captures the valorization of fermentation residues into UERM, supporting yeast cultivation and reducing dependency on animal- or plant-derived nitrogen sources. The second loop highlights the carbon flow, where Ulva acts as a CO_2_ sink during growth and a CO_2_ source during fermentation, reflecting a near-balanced biogenic carbon cycle. This integrated approach not only minimizes process waste but also aligns with global objectives for circular bioeconomy and low-carbon transitions. Such system-level strategies offer a replicable model for marine biorefineries seeking both ecological restoration and industrial sustainability.

## 5. Conclusions

In conclusion, this study demonstrated the feasibility of converting seasonally abundant *U. ohnoi* biomass into bioethanol and microbial culture media using an integrated biorefinery approach. Seasonal variations influenced the biochemical composition and saccharification efficiency of the biomass, with spring and winter samples showing the highest sugar yield and ethanol productivity. Post-fermentation residues were successfully valorized in UERM containing functional levels of amino acids that supported yeast growth and fermentation performance equivalent to the conventional YPD medium. Despite minor deficiencies in specific amino acids, UERM was metabolically sufficient, highlighting its potential as a low-cost and sustainable nitrogen source. Our findings provide a practical nature-based strategy for addressing *Ulva* overgrowth by transforming excess biomass into renewable bioresources. By aligning seasonal bloom cycles with biotechnological valorization, such as bioethanol production and microbial media development, this approach supports coastal ecosystem restoration while advancing circular bioeconomy principles.

## Figures and Tables

**Table 1:** Operating conditions of amino acid analysis.

	Amino Acids	Free Amino Acid
Instrument	Agilent 1200 LC	Dionex Ultimate 3000
FL detector	Emission 450 nm, Excitation 340 nm (OPA) Emission 305 nm, Excitation 266 nm (FMOC)	Emission 450 nm, Excitation 340 nm (OPA) Emission 305 nm, Excitation 266 nm (FMOC)
UV detector	338 nm	338 nm
Column	C18 column (4.6 mm × 150 mm, 5 µm)	Inno C18 column (4.6 mm × 150 mm, 5 µm/YoungJin biochrom, Republic of Korea)
Column temperature	40 °C	40 °C
Injection volume	0.5 µL	0.5 µL
Mobile phase	A: ACN/MeOH/DW = 9/9/1 B: 40 mM Phosphate buffer (pH 7.5)	Mobile Phase A: 40 mM Sodium phosphate dibasic, pH 7 Mobile Phase B: 3 DW/Acetonitrile/Methanol (10:45:45 *v/v*%)

**Table 2:** Seasonal biochemical composition of *Ulva ohnoi* collected from Sinyang, Jeju.

	Spring	Summer	Autumn	Winter
Protein (%)	14.49 ± 2.56	16.10 ± 1.81	11.62 ± 0.56	21.64 ± 1.87
Carbohydrates (%)	55.35 ± 3.04	45.74 ± 3.90	53.31 ± 2.40	50.39 ± 1.58
Lipid (%)	0.81 ± 0.95	1.07 ± 0.15	0.73 ± 0.16	1.02 ± 0.82
Ash (%)	20.04 ± 2.55	29.14 ± 3.95	26.24 ± 2.24	20.35 ± 3.13
Moisture (%)	9.31 ± 1.99	7.94 ± 0.32	8.10 ± 0.70	6.60 ± 1.73

**Table 3:** Relative proportions of free amino acids in *Ulva* ethanol residue medium (UERM).

Free Amino Acid	Spring	Summer	Autumn	Winter
Asparagine	0.09	0.09	0	0
Tyrosine	0.37 ± 0.01	0.39	0.35	0.36
Serine	0	0.09	0	0
Glutamic acid	0.38 ± 0.01	0.20 ± 0.01	0.27 ± 0.01	0.32
Proline	1.20 ± 0.04	0.81 ± 0.08	1.02 ± 0.05	1.04 ± 0.05
Glycine	0.47	0.37 ± 0.01	0.47 ± 0.05	0.51 ± 0.05
Alanine	1.57	1.30 ± 0.02	1.46 ± 0.01	1.52
Valine	1.31 ± 0.01	1.07 ± 0.01	1.21 ± 0.01	1.23 ± 0.05
Isoleucine	0.32	0.30	0.30	0.30
Leucine	0	0	0	0
Tyrosine	0.37 ± 0.01	0.36	0.35	0.36
Phenylalanine	0.70	0.66 ± 0.01	0.63	0.65 ± 0.01
Histidine	0	0	0	0
Lysine	0.70	0.51 ± 0.02	0.70	0.71
Arginine	0.88	0.72	0.91 ± 0.01	0.94
Cystein	0	0	0	0
Methionine	0.68	0.46	0.34 ± 0.01	0.37
Tryptopan	0	0	0	0
Total	9.02 ± 0.01	7.31 ± 0.01	8.01 ± 0.01	8.30 ± 0.02

**Table 4:** Seasonal variations in the fermentaion performance of UERM-grown yeast cultured on *Ulva ohnoi* hydrolysate.

	Reducing Sugar		Ethanol	Y_EtOH_
	0 h	24 h	24 h	
Spring	36.24 ± 0.24	0.22 ± 0.24	16.83 ± 0.80	0.46
Summer	29.52 ± 0.32	0.12 ± 0.17	12.94 ± 0.45	0.44
Autumn	35.01 ± 0.35	0.22 ± 0.54	15.73 ± 0.22	0.45
Winter	36.50 ± 0.02	0.12 ± 0.35	16.52 ± 0.12	0.45

## Data Availability

All authors of articles published in MDPI journals to share the research data.
